# Assessment of the Role of Endometrial Receptivity Analysis in Enhancing Assisted Reproductive Technology Outcomes for Advanced-Age Patients

**DOI:** 10.7759/cureus.62949

**Published:** 2024-06-23

**Authors:** Tamar Barbakadze, Mariam Shervashidze, Tea Charkviani, Tengiz Zhorzholadze, Tamar Kbilashvili, Mariam Gabadze, Tea Pataraia, Ana Pantskhava, Zeinab Beridze, Jenara Kristesashvili

**Affiliations:** 1 Faculty of Clinical and Translational Medicine, Ivane Javakhishvili Tbilisi State University, Tbilisi, GEO; 2 Department of Reproductive Endocrinology, ReproArt Georgian-American Center for Reproductive Medicine, Tbilisi, GEO; 3 Department of Embryology, ReproArt Georgian-American Center for Reproductive Medicine, Tbilisi, GEO; 4 Department of Reproductive Endocrinology, ReproArt Georgian-American Center for Reproductive Medicine, Kutaisi, GEO; 5 Department of Reproductive Endocrinology, ReproArt Georgian-American Center for Reproductive Medicine, Batumi, GEO

**Keywords:** recurrent implantation failure (rif), endometrial receptivity analysis (era), aging of the endometrium, oocyte donation, preimplantation genetic testing for aneuploidy (pgt-a)

## Abstract

Background and objectives: In contemporary society, socially active women are increasingly planning their fertility for later in life. The fertility outcomes for advanced-age patients, even with egg donation, are often suboptimal due to endometrial aging. Recurrent implantation failure (RIF) is one of the core problems for assisted reproductive technology (ART), especially for advanced-age patients. High-quality, euploid embryos and synchronization between the embryonic stage and the uterine endometrial lining are crucial for positive outcomes. The study aims to improve ART outcomes with personalized embryo transfer (pET) according to endometrial receptivity analysis (ERA) in advanced-age patients with challenging reproductive histories, and RIF by utilizing, donor oocytes and preimplantation genetic testing for aneuploidy (PGT-A) for embryo testing.

Methods: A randomized, controlled observational follow-up study was conducted from 2020 to 2023. After obtaining informed consent, 320 patients with RIF were selected. Patients were allocated into the study group and control group 1 based on consistent application of randomization principles, while control group 2 was selected separately. The study group included patients undergoing PGT-A and ERA, aged 35-45 years, with a mean age of 40.5±3.7 years. Control group 1 comprised patients undergoing PGT-A, aged 35-45 years, with a mean age of 40±4.2 years. Control group 2 consisted of patients undergoing PGT-A and ERA, aged less than 35 years, with a mean age of 31.6±2.2 years.

Results: Results suggest that ERA may improve implantation and pregnancy outcomes in advanced-age patients, particularly those with RIFs. The pregnancy rate was significantly higher in the study group (77.9%), compared to control group 1 (57.6%) (p=0.0007), and no significant difference compared to control group 2 (77.3%) (p=0.94). The implantation rate was higher in the study group (54.1%) than in control group 1 (39.4%) (p=0.0009), and there was no significant difference between the study group and control group 2 (50%, p=0.87). The live birth rate was also higher in the study group (71.3%), compared to control group 1 (39.4%) (p<0.0001). There were no significant differences between the study group and control group 2 (65.9%, p=0.50).

Conclusion: pET guided by ERA significantly improves pregnancy, implantation, and live birth rates in advanced-age patients with challenging reproductive histories. pET provides ART outcomes with no significant difference between advanced-age patients and younger patients with pET guided by ERA.

## Introduction

Contemporary lifestyle modifications have postponed conception, rendering advanced maternal age a significant risk factor for female infertility [1]. It is well substantiated that advancing maternal age strongly correlates with deteriorating oocyte quality and increased risk of chromosomal abnormalities in oocytes and embryos. However, the reduction in fertility with age is not only due to ovarian causes. Advances in assisted reproductive technology (ART), such as oocyte donation and the selection of viable embryos through preimplantation genetic testing for chromosomal abnormalities (PGT-A), have addressed several ovarian-related difficulties associated with advanced maternal age. Nevertheless, other variables, particularly endometrial aging, have a significant influence on implantation rates, clinical pregnancy rates, and live birth rates, as well as overall female fertility in women of advanced age [2].

The clinical relevance of the endometrial receptivity analysis (ERA) resides in its ability to guide personalized embryo transfer (pET) by aligning the embryo transfer with the patient's distinctive window of implantation (WOI). This synchronization proves particularly beneficial for patients encountering implantation failure due to endometrial factors. By leveraging ERA, clinicians can tailor the timing of embryo transfer to coincide with the individual's endometrial receptivity profile, thereby optimizing the probability of successful implantation through a personalized approach and ultimately enhancing overall reproductive outcomes. In patients of advanced age, achieving comparable outcomes to those seen in patients of younger reproductive age is feasible [3].

Nonetheless, the lack of published studies on this topic shows that the field currently needs to be explored. Enhancing our understanding of methods improves ART in advanced maternal age.

The study aims to improve ART outcomes with pET according to ERA in advanced-age patients with challenging reproductive histories and recurrent implantation failure (RIF) by utilizing donor oocytes and PGT-A for embryo testing.

## Materials and methods

Study design

A prospective, randomized, observational follow-up study was conducted from March 2020 to September 2023 at ReproArt Georgian-American Center for Reproductive Medicine. Patients were allocated to the study and control groups based on the principle of randomization and application consistency. Obstetrical and neonatal outcomes were systematically documented, with data on pregnancy outcomes collected from a secure electronic national registry.

Ethical considerations

All procedures performed in the study were in accordance with the ethical standards of the institutional and national research committee and with the 1964 Helsinki Declaration and its later amendments or comparable ethical standards. The study protocol and a draft consent agreement for participation in the study were approved by the Ethics Committee of the Institution Review Board of ReproArt Georgian-American Center for Reproductive Medicine (#2-20/287; February 7; 2020). Informed consent was obtained from all individual participants included in the study.

Study criteria

Infertile patients undergoing ART were divided into groups based on the source of oocytes: donor oocytes were used for the study group and control group 1, while patients in control group 2 utilized their own oocytes. Patients who had experienced RIF with euploid embryo transfers and had at least one frozen euploid embryo were included. Study participants were selected after obtaining informed consent. They were allocated into study and control groups based on consistent application and randomization principles. The inclusion criteria were as follows: the age range for the study group and control group 1 was 35-45 years, while for control group 2 it was 28-34 years. Patients must have had frozen euploid blastocysts (developed to day 5/6) analyzed by PGT-A. In the study group and control group 1, patients had embryos obtained from donor oocytes fertilized via in vitro fertilization (IVF)/intracytoplasmic sperm injection (ICSI), whereas in control group 2, patients had embryos obtained from their own oocytes fertilized via IVF/ICSI. The expected embryo transfer involved one or two embryos (single embryo transfer (SET) or double embryo transfer (DET)) in a hormonal replacement therapy (HRT) cycle. The body mass index (BMI) of patients ranged from 18.5 to 30 kg/m².

The exclusion criteria for the study included the presence of uterine cavity pathologies or malformations, such as polyps, intramural myomas of 4 cm or larger, submucosal myomas, septum, or hydrosalpinx, identified during the patient's participation in the study. However, patients diagnosed with these conditions before or after inclusion were permitted to participate if the pathology was corrected prior to any study procedures. Additionally, any illness or medical condition deemed unstable or that, according to medical judgment, could compromise the patient's safety and compliance with the study, was grounds for exclusion.

After the patients had at least one euploid embryo, they were suitable for randomization into the study group and control group 1 and for selection in control group 2.

Procedure

After normal basal ultrasound, from day 2-3 after menstruation controlled ovarian stimulation was done using 375 IU recombinant FSH, with 75 IU human menopausal gonadotrophin (HMG) for the first two days, followed by 150 IU recombinant FSH and 75 IU HMG from the third day of ovarian stimulation. As for the trigger, gonadotropin-releasing hormone agonist GnRH agonist 0.2mg/2ml and human chorionic gonadotropin (HCG )1500 IU were used. Transvaginal ultrasound examination, serum oestradiol (E2), FSH, and luteinizing hormone (LH) determination were started from the day of ovarian stimulation and repeated every 48 hours. The average duration of ovarian stimulation was 10- 12 days. Oocyte retrieval was done after 36 hours after trigger injection. ICSI was carried out, and fertilization was assessed after 17/20 hours after microinjection. Embryos were cultured according to IVF laboratory protocol. Embryo quality was evaluated according to Garndner’s criteria [4]. On day 5/6 of embryo development, a trophectoderm biopsy was done. Embryos were frozen according to the IVF laboratory protocol, and biopsied samples were sent for genetic testing for aneuploidy. Samples were analyzed utilizing next-generation sequencing. Only euploid embryos were selected for transfer.

In the study group and control group 1, the patient underwent one or two endometrial biopsies, and embryo transfer was done in an HRT cycle at the timing indicated by the ERA test.

Endometrial biopsies were collected from the uterine fundus using a Pipelle catheter or office hysteroscopy under sterile conditions. After the biopsy, the endometrium tissue was transferred to a cryotube containing 1.5ml of ribonucleic acid Later (RNALater), vigorously shaken for a few seconds, and kept at 4^o^C or in ice for at least 4h. The samples were shipped at room temperature for the ERA test to Igenomix.

The endometrium was prepared for the ERA, pET, and frozen embryo transfer cycles using HRT. On the second or third day of menstruation, a vaginal ultrasound was performed, and blood levels of estradiol and progesterone (P4) were measured. Patients received 6 mg of oral oestradiol and 3 grams of transdermal oestradiol daily. Sonographic evaluations and E2 and P4 assessments were conducted between 7 and 12 days after endometrial preparation. The mean endometrial thickness was 8.7 mm (standard deviation (SD) 0.92). On the day of progesterone introduction, P4 levels were <1 ng/mL. For luteal phase support, patients received 600 mg of vaginal progesterone and 20 mg of dydrogesterone per day.

In control group 1, embryo transfer was performed approximately 120 hours after progesterone introduction. For the study group and control group 2, embryo transfer was conducted according to the ERA-recommended period. In all three groups, high-quality euploid embryos were transferred, with either SET or DET performed. To avoid bias the quantity of transferred embryos did not differ significantly.

Assessments

Outcomes were measured based on the pregnancy rate, implantation rate, and live birth rate.

Statistical analysis

The reproductive outcomes at first embryo transfer during one-year follow-up in the study and control groups were assessed through intention-to-treat analysis. The results of the study were treated statistically using the software IBM SPSS Statistics for Windows, Version 22 (Released 2013; IBM Corp., Armonk, New York, United States). Continuous variables were expressed as mean SD. The comparison of these parameters between groups was performed by an independent t-test. Categorical variables were expressed as percentages. The comparison of these parameters between groups was performed by the independent t-test. The comparison of these parameters between groups was performed using the odds ratio (OR) and 95% confidence interval (95% CI). p-values of <0.05 were considered to indicate statistical significance.

## Results

Between March 2020 and September 2023, a total of 320 patients were recruited after meeting the inclusion criteria and providing informed consent. The study group consisted of 138 patients, control group 1 consisted of 140 patients, and control group 2 consisted of 42 patients. Following the follow-up period, 16 patients were excluded from the study group due to endometrial thickness <6 mm or progesterone levels >1 ng/ml; eight patients were excluded from control group 1 for the same reasons. Additionally, six patients from control group 2 experienced spontaneous pregnancy before starting the cycle for embryo transfer. Consequently, 122 patients in the study group, 132 in control group 1, and 36 in control group 2 completed all procedures included in the study protocol.

Baseline demographics, family history, personal history, harmful habits, previous surgeries, and clinical characteristics were comparable among the groups. The follicle-stimulating hormone (FSH) and anti-Müllerian hormone (AMH) values of egg donors were similar between the study group and control group 1. However, in control group 2, the FSH and AMH values showed slight variations compared to the egg donor group. Cycle characteristics and embryological data were broadly consistent across the groups, with no significant differences observed.

A comparison of the reproductive parameters means between the study group and control group 1 showed no statistically significant differences (Table 1).

**Table 1:** Reproductive Parameters: Comparison of Means in the Study and Control Groups FSH: Follicle-stimulating hormone; AMH: anti-Müllerian hormone; AFC: antral follicle count; MII: metaphase II; PGT-A: preimplantation genetic testing for aneuploidy; ERA: endometrial receptivity analysis; SD: standard deviation

Parameter	PGT-A+ERA (n=122)	PGT-A (n=132)	PGT-A+ERA vs. PGT-A	
	Mean ±SD	Mean ±SD	Independent t-test	p-value
Egg Donor Age	23.4±3.7	23.4±3.7	1.850	0.066
Egg Donor Basal FSH	6.7 ± 1.3	6.1 ±3.3	1.878	0.061
Egg Donor AMH	5.8± 2.0	5.3± 2.3	1.842	0.066
Sperm Concentration	82.3± 51.7	75.1± 45.2	1.184	0.238
Egg Donor AFC	26.6 ± 8.4	24.8 ± 9.0	1.644	0.101
Egg Donor M II	16.7 ± 5.9	15.9 ± 5.8	1.634	0.104
Fertilization Rate - Fertilized Oocytes/M II	0.89 ± 0.10	0.90± 0.22	0.460	0.646
Blastocyst Formation Rate - Vitrified Embryos/Fertilized Oocytes	0.62± 0.19	0.59 ± 0.19	1.257	0.210

A comparison of reproductive parameters means such as basal FSH, AMH, antral follicle count (AFC), and blastocyst formation rate showed a statistically significant difference between the study group and control group 2. No statistically significant difference was observed for the parameters sperm concentration and fertilization rate (Table 2).

**Table 2:** Reproductive Parameters: Comparison of Means in the Study and PGT-A+ERA Groups According to the Patient Age FSH: Follicle-stimulating hormone; AMH: anti-Müllerian hormone; AFC: antral follicle count; MII: metaphase II; PGT-A: preimplantation genetic testing for aneuploidy; ERA: endometrial receptivity analysis; SD: standard deviation

Parameter	PGT-A+ERA Age 35years (n=122)	PGT-A+ERA Age<35years (n=44)	PGT-A+ERA vs. PGT-A	
	Mean ± SD	Mean± SD	Independent t-test	p-value
Egg Donor Age	23.4±3.7	28 ± 4.2	13.96	0.001
Egg Donor Basal FSH	6.7 ±1.3		2.478	0.014
Patient Basal FSH		7.3 ± 1.5		
Egg Donor AMH	5.8 ± 2.0		10.026	<0.001
Patient AMH		2.3± 1.8		
Sperm Concentration	82.3 ± 51.7	71.8 ± 58.4	1.098	0.274
Egg Donor AFC	26.6 ± 8.4		8.130	<0.001
Patient AFC		14.8 ± 7.2		
Egg Donor M II	16.7 ± 4.9		5.543	<0.001
Patient M II		11.6 ± 5.8		
Fertilization Rate - Fertilized Oocytes/M II	0.89 ± 0.10	0.89 ± 0.08	0.059	0.953
Blastocyst Formation Rate - Vitrified Embryos/Fertilized Oocytes	0.62± 0.19	0.50 ± 0.14	3.754	<0.001

According to the intention-to-treat analysis, no statistically significant difference was found between the outcomes. The pregnancy rate was statistically significantly higher in the study group in comparison with the control group (p=0.0007). The implantation rate was statistically significantly higher in the study group compared to control group 1 (p=0.0009). The live birth rate was statistically significantly higher in the study group in comparison with control group 1 (p<0.0001). No statistically significant difference was found between the study group and control group 1 in biochemical pregnancy (p=0.90) and clinical miscarriages (p=0.065) (Table 3).

**Table 3:** Reproductive Outcomes at First Embryo Transfer During One-Year Follow-Up: Intention-to-Treat Analysis in the Study and Control Group 1 PGT-A: Preimplantation genetic testing for aneuploidy; ERA: endometrial receptivity analysis; IUGR: intrauterine growth restriction; CI: confidence interval

Outcome	PGT-A+ERA (n=122)	PGT-A (n=132)	PGT-A+ERA vs. PGT-A	
			Odds Ratio (95%CI)	p-value
Transfers, n	122	132		
Pregnancy rate, n (%)	95 (77.9%)	76 (57.6%)	2.59 (1.50-4.49)	0.0007
Implantation rate, n (%)	114/223 (54.1%)	78/220 (35.5%)	1.90 (1.30-2.79)	0.0009
Live birth rate n (%)	87 (71.3%)	52 (39.4%)	3.82 (2.26-6.47)	<0.0001
Singleton	63 (72.4%)	36 (69.3%)	1.17 (0.55-2.48)	0.69
Multiple (all twins)	24 (27.6%)	16 (30.7%)	0.86 (0.40-1.82)	0.69
Clinical miscarriages, n (%)	3/95 (3.2%)	8/76 (10.5%)	0.27 (0.07-1.08)	0.065
Biochemical pregnancies, n (%)	5/95 (5.3%)	10/76 (13.2%)	0.37 (0.12-1.12)	0.090
Ectopic pregnancies, n (%)	0/95 (0.0%)	0/76 (0.0%)	N/A	N/A
Neonatal mortality, n (%)	0/95 (0.0%)	0/76 (0.0%)	N/A	N/A
Preeclampsia, n (%)	6/95 (6.3%)	0/76 (0.0%)	N/A	N/A
IUGR	0/95 (0.0%)	0/76 (0.0%)	N/A	N/A
Metabolic disorders	0/95 (0.0%)	0/76 (0.0%)	N/A	N/A
Infections	0/95 (0.0%)	0/76 (0.0%)	N/A	N/A
Gestational diabetes	0/95 (0.0%)	2/76 (2.6%)	N/A	N/A
1^st^ trimester bleeding	36/95 (37.9%)	18/76 (23.7%)	1.85 (0.93-3.67)	0.078
3^rd^ trimester bleeding	0/95 (0.0%)	0/76 (0.0%)	N/A	N/A

According to the intention-to-treat analysis, no statistically significant difference was found between the study group and control group 2 in terms of pregnancy rate, implantation rate, live birth rate, biochemical pregnancy, and clinical miscarriages (Table 4).

**Table 4:** Reproductive Outcomes at First Embryo Transfer During One-Year Follow-Up: Intention-to-Treat Analysis in the Study and Control Group 2 PGT-A: Preimplantation genetic testing for aneuploidy; ERA: endometrial receptivity analysis; IUGR: intrauterine growth restriction; CI: confidence interval

Outcome	PGT-A+ERA Age 35years (n=122)	PGT-A+ERA Age<35years (n=44)	PGT-A+ERA 35 years vs. PGT-A <35 years	
			Odds Ratio (95%CI)	p-value
Transfers, n	122	44		
Pregnancy rate, n (%)	95 (77.9%)	34 (77.3%)	1.03 (0.45-2.36)	0.94
Implantation rate, n (%)	114/223 (54.1%)	36/72 (50.0%)	1.05 (0.61-1.78)	0.87
Live birth rate n (%)	87 (71.3%)	29 (65.9%)	1.29 (0.62-2.69)	0.50
Singleton	63 (72.4%)	24 (82.8%)	1.17 (0.55-2.48)	0.69
Multiple (all twins)	24 (27.6%)	5 (17.2%)	0.55 (0.19-1.60)	0.27
Clinical miscarriages, n (%)	3/95 (3.2%)	2/29 (6.9%)	1.83 (0.63-5.34)	0.27
Biochemical pregnancies, n (%)	5/95 (5.3%)	0/29 (0.0%)	N/A	N/A
Ectopic pregnancies, n (%)	0/95 (0.0%)	0/29 (0.0%)	N/A	N/A
Neonatal mortality, n (%)	0/95 (0.0%)	0/29 (0.0%)	N/A	N/A
Preeclampsia, n (%)	6/95 (6.3%)	0/29 (0.0%)	N/A	N/A
IUGR	0/95 (0.0%)	0/29 (0.0%)	N/A	N/A
Metabolic Disorders	0/95 (0.0%)	0/29 (0.0%)	N/A	N/A
Infections	0/95 (0.0%)	0/29 (0.0%)	N/A	N/A
Gestational Diabetes	0/95 (0.0%)	0/29 (0.0%)	N/A	N/A
1^st^ trimester Bleeding	36/95 (37.9%)	0/29 (0.0%)	N/A	N/A
3^rd^ trimester Bleeding	0/95 (0.0%)	0/29 (0.0%)	N/A	N/A

**Figure 1:**
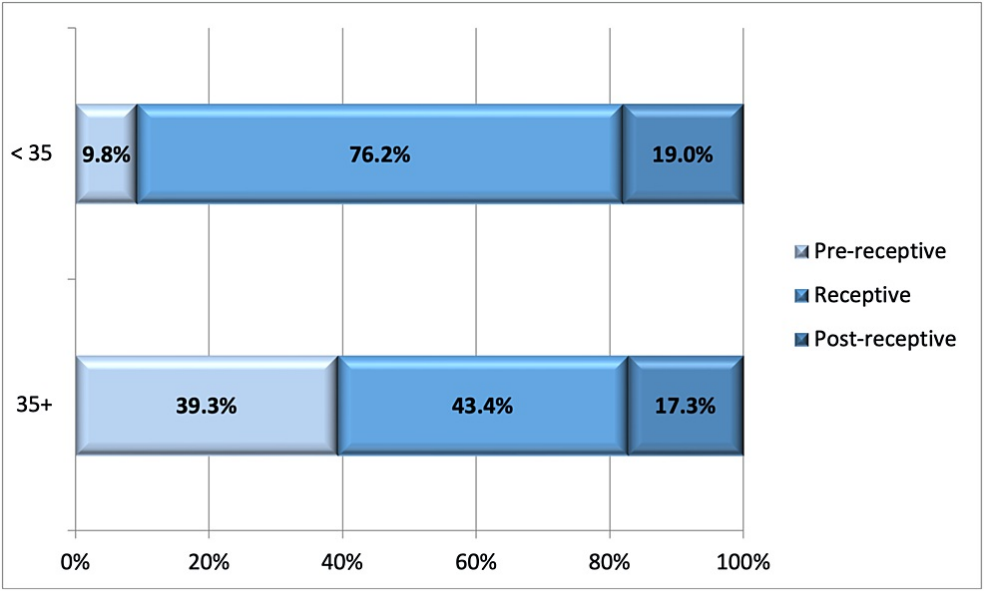
Rate and Type of Endometrial Receptivity Displacement in Advanced-Age Patients Compared to the Younger Age Group

The study results indicate that the WOI was adjusted more frequently in advanced-age patients compared to the younger age group. There was no statistically significant difference in early endometrial receptivity between the two groups. However, late endometrial receptivity was more prevalent among advanced-age patients (Figure 1).

## Discussion

Our study included patients with euploid embryos to avoid potential biases from embryonic factors. Our opinion in this regard coincides with the results of Munné’s research, which shows that even with egg donation, there is a risk of aneuploid embryos; in donor cycles, 68.8±22.2% were euploid, as reported by Munné et al. [5].

Our research demonstrates statistically significant outcomes, in particular, 95 pregnancies from 122 embryo transfers in the study group, compared to 76 pregnancies from 132 embryo transfers in control group 1. This clearly shows the advantages of pET guided by the ERA. The implantation rates were in the study group and control group 1. The live birth rates in the PGT-A+ERA group were 39.4%. Our research demonstrates statistically significant outcomes, particularly 95 pregnancies from 122 embryo transfers (77.9%) in the study group, compared to 76 pregnancies from 132 embryo transfers (57.6%) in control group 1. This clearly shows the advantages of pET guided by the ERA. The implantation rates were 54.1% in the study group and 35.5% in control group 1. The live birth rates were 71.3% in the PGT-A+ERA group and 39.4% in the PGT-A group. These outcomes indicate that ERA not only supports successful implantation but also ensures normal implantation and decidualization leading to lower pregnancy complications and a lower clinical miscarriage rate in the PGT-A+ERA group [6]. Furthermore, a randomized controlled trial (RCT) by Simon et al. demonstrated statistically significant improvements in pregnancy, implantation, and cumulative live birth rates with pET compared to frozen and fresh embryo transfers [7].

The comparison between our study group and control group 2 showed similar success rates, with higher live birth rates in the >35 PGT-A+ERA group. Despite the relationship between advanced maternal age and endometrial receptivity, it is multifaceted, encompassing hormonal, cellular, and molecular dimensions [8]. Recent studies, including ours, have sought to elucidate these complexities, particularly how aging impacts endometrial function and fertility outcomes [9].

Further analysis of the ERA results revealed that in advanced-age patients, the WOI was more frequently altered compared to younger patients, with late receptivity being more prevalent in this demographic [10]. These findings underscore the clinical relevance of ERA in tailoring ART procedures to individual endometrial profiles, thereby maximizing the likelihood of successful pregnancies. Moreover, the comparison between the study group and control group 2, consisting of younger patients, showed no significant difference in reproductive outcomes, suggesting that ERA-guided pET can elevate the reproductive success rates of advanced-age patients to levels comparable to those of younger patients. The data implies that the critical determinant of successful outcomes is the alignment of embryo transfer with the individual's WOI rather than the patient's chronological age. However, it is worth noting that the study design similar to ours needs to be more broadly described in the scientific literature, and the generalizability of these results to other study populations in different countries remains to be explored. pET, according to ERA, in the>35 age group patients provides a successful outcome of ART as in the <35 age group.

Contrary to reports suggesting that endometrial aging leads to decreased receptivity, our findings align with those of Paulson et al. [11] indicating that endometrial receptivity might remain stable with age, particularly with high-quality embryos. Our results showed displaced receptivity rates of 23.8% in patients <35 years and 56.6% in those >35 years, underscoring the importance of ERA in advanced reproductive age. The ERA proved critical for optimizing transfer timing and provides no significant differences in clinical outcomes between these age groups.

According to study results by Moreno et al., the current understanding of endometrial aging also underscores the role of the microbiome in reproductive health. Recent studies indicate that a dysbiotic microbiome in the endometrium is associated with the pre-receptive endometrium and could disrupt the WOI and fertility outcome. This finding highlights the need for a holistic approach to addressing age-related infertility, considering both the microbiome and traditional hormonal and cellular markers [12]. Our study demonstrated significant improvement even with ERA.

Moreover, advanced molecular techniques like the ERA have shown promise in improving outcomes for patients with RIF. These tools help accurately determine the WOI and synchronize embryo transfer with the endometrium's receptive state, enhancing the chances of successful implantation and pregnancy [13].

Our study demonstrates that pET guided by ERA significantly improves ART outcomes in advanced-age patients. The pregnancy rate, implantation rate, and live birth rate were significantly higher in the study group compared to control group 1, highlighting the efficacy of ERA in optimizing reproductive outcomes. The live birth rate in the study group was almost double that of control group 1, indicating a substantial improvement in outcomes through personalized approaches [14].

In summary, our study highlights the critical role of personalized embryo transfer guided by ERA in improving ART outcomes for advanced-age patients. By aligning embryo transfer with the patient's unique WOI, clinicians can enhance implantation success rates, leading to higher pregnancy and live birth rates [15,16]. Future research should focus on refining these personalized approaches and exploring additional factors influencing endometrial receptivity, such as the microbiome, to optimize reproductive outcomes for all ART patients [17].

The cost-effectiveness of ERA and pET has been discussed in reproductive medicine. ERA is designed to identify the optimal time for embryo transfer by analyzing gene expression in endometrial tissue. Studies indicate that while ERA-guided pET may improve implantation and live birth rates, the higher procedural costs can impact cost-effectiveness. For instance, the study showed that fresh embryo transfer had the lowest cost per live birth, while pET required a higher live birth rate to be cost-effective compared to standard methods [18].

ERA is designed to identify the optimal time for embryo transfer by analyzing gene expression in endometrial tissue. Studies indicate that while ERA-guided pET may improve implantation and live birth rates, the higher procedural costs can impact cost-effectiveness. For instance, the study showed that fresh embryo transfer had the lowest cost per live birth, while pET required a higher live birth rate to be cost-effective compared to standard methods.

A limitation of our study may be the recruitment of participants for RCTs. The high cost of ART, which is not covered by insurance, presents substantial challenges in attracting sufficient participants. This financial barrier may impact the generalizability and scalability of our findings. Further RCTs would be more informative to increase the number of patients in the age group <35 and to broadly compare the outcomes of PGT-A tested embryo transfers using the conventional approach versus personalized embryo transfer according to the ERA. However, challenges such as postponing fertility for advanced reproductive age and delayed referral to fertility specialists remain significant obstacles.

## Conclusions

The incorporation of pET guided by ERA markedly improves ART outcomes, allowing reproductive specialists to achieve success rates in older patients that rival those of younger individuals. By utilizing cutting-edge molecular biology techniques, ERA not only reduces the time needed for reproductive interventions but also addresses the complexities of endometrial receptivity, which is influenced by hormonal fluctuations and the endometrial microbiome. Integrating molecular diagnostics such as ERA into clinical practice holds great promise for personalized reproductive medicine, particularly benefiting older women and those experiencing RIF. Future RCTs are crucial to further refine these methods and develop targeted treatments to counteract endometrial aging, ultimately improving ART outcomes for all age groups. For future research, we suggest focusing on diverse patient populations, including various age groups and reproductive health conditions, to understand the applicability and effectiveness of ERA and pET. Additionally, age-specific studies, research on heterogeneous reproductive health conditions, longitudinal studies to track long-term outcomes, and comparative studies between traditional and personalized fertility treatments are essential to enhance and tailor fertility treatments effectively.
